# The effect of ambient temperature on type-2-diabetes: case-crossover analysis of 4+ million GP consultations across England

**DOI:** 10.1186/s12940-017-0284-7

**Published:** 2017-07-12

**Authors:** S. Hajat, A. Haines, C. Sarran, A. Sharma, C. Bates, L. E. Fleming

**Affiliations:** 1 London School of Hygiene & Tropical Medicine, London, UK; 2 Met Office, Exeter, UK; 3 TPP, Horsforth, Leeds, UK; 4 University of Exeter Medical School, Truro, UK; 5 Department of Social & Environmental Health Research, London School of Hygiene & Tropical Medicine, 15-17 Tavistock Place, London, WC1H 9SH UK

**Keywords:** Climate change, Weather, Diabetes mellitus, Primary care

## Abstract

**Background:**

Given the double jeopardy of global increases in rates of obesity and climate change, it is increasingly important to recognise the dangers posed to diabetic patients during periods of extreme weather. We aimed to characterise the associations between ambient temperature and general medical practitioner consultations made by a cohort of type-2 diabetic patients. Evidence on the effects of temperature variation in the primary care setting is currently limited.

**Methods:**

Case-crossover analysis of 4,474,943 consultations in England during 2012–2014, linked to localised temperature at place of residence for each patient. Conditional logistic regression was used to assess associations between each temperature-related consultation and control days matched on day-of-week.

**Results:**

There was an increased odds of seeking medical consultation associated with high temperatures: Odds ratio (OR) = 1.097 (95% confidence interval = 1.041, 1.156) per 1 °C increase above 22 °C. Odds during low temperatures below 0 °C were also significantly raised: OR = 1.024 (1.019, 1.030). Heat-related consultations were particularly high among diabetics with cardiovascular comorbidities: OR = 1.171 (1.031, 1.331), but there was no heightened risk with renal failure or neuropathy comorbidities. Surprisingly, lower odds of heat-related consultation were associated with the use of diuretics, anticholinergics, antipsychotics or antidepressants compared to non-use, especially among those with cardiovascular comorbidities, although differences were not statistically significant.

**Conclusions:**

Type-2 diabetic patients are at increased odds of medical consultation during days of temperature extremes, especially during hot weather. The common assumption that certain medication use heightens the risk of heat illness was not borne-out by our study on diabetics in a primary care setting and such advice may need to be reconsidered in heat protection plans.

**Electronic supplementary material:**

The online version of this article (doi:10.1186/s12940-017-0284-7) contains supplementary material, which is available to authorized users.

## Background

It is commonly recognised that temperature extremes raise the risk of mortality and morbidity from cardio-respiratory conditions, and diabetic patients are also at increased risk. For example, in the US diabetics were reported to have a 17% higher risk of dying on hot days compared to other subjects, which was greater than for any other disease considered [1]. This is probably due to compromised heat dissipation among diabetics which increases the risk of heat-related illness [2, 3]. Furthermore, the condition can also lead to impaired vascular response similar to that commonly observed among the elderly during cold weather conditions [4]. The global prevalence of diabetes among adults rose from 4.7% in 1980 to 8.5% (422 million people) in 2014, of which about 90% have type-2 diabetes which is largely caused by excess body weight and a sedentary lifestyle [5]. The World Health Organization projects that diabetes will be the 7th leading cause of death globally by 2030 [6].

Given the double jeopardy of global increases in obesity rates and global climate change, it is increasingly important to recognise the dangers posed to diabetic patients during periods of extreme weather, especially high temperatures. Although such individuals may be identified as high-risk in the public health heat-protection plans of many countries, the unique challenges faced by diabetics are rarely addressed. For example, since type-2 diabetes is increasingly diagnosed in younger adults and children due to rising obesity rates in these groups, impacts may not be restricted to older age-groups traditionally at risk during hot and cold weather. Comorbidities commonly associated with diabetes, such as chronic kidney disease, cardiovascular disease and neurologic sequelae, may confer additional risks during climate extremes [7]. Furthermore, medications frequently prescribed to diabetics such as diuretics and salicylates can adversely affect thermoregulation, and the use of other common drugs such as anticholinergics, antidepressants and antipsychotics that interfere with the normal sweating process may also intensify heat-related risk in this sensitive group [7]. Although mechanisms have been postulated by which medications may heighten heat risk, there is little epidemiological evidence to support singling-out specific drug-types, nor the extent to which any increased risk is attributable to the drug or to the underlying disease it is being used to treat [8].

Evidence on the effects of heat and cold exposure in the primary care setting are limited compared to other morbidity outcomes such as hospitalisations, even though the number of patient contacts involved is greater and intervention at this stage has the potential to prevent heat-related illness deteriorating, resulting in hospitalisation or death. Use of general medical practitioner (GP) patient data also opens up the possibility of assessing important comorbidities and information on medication use which may not be available with other databases.

Another limitation of much previous work is the characterization of ambient temperature exposure based on measurements recorded at fixed monitoring stations, which may not be a good indicator of personal exposure. Gridded climate datasets based on the interpolation of observed measurements can determine weather conditions at high spatial resolution and so have the potential to overcome this problem [9].

In this study we linked the place of residence of a large cohort of type-2 diabetic patients registered on GP databases across England to gridded climate data in order to characterise temperature-related consultations among these high-risk patients, and to assess possible modification of effects by patient characteristics, comorbidities and medication use. This information has the potential to identify and provide support for those diabetic patients and their healthcare providers most at risk during climate extremes.

## Methods

### Health data

General practice data were obtained from the ResearchOne database developed by TPP (The Phoenix Partnership) in partnership with the University of Leeds and the UK Government’s Technology Strategy Board (http://www.researchone.org). The database consists of de-identified clinical and administrative data drawn from electronic patient records held on the TPP ‘SystmOne’ clinical system.

Based on the Read Codes (Clinical Terms Version 3) listed in Additional file 1: Table S1, 191,842 type-2 diabetic patients were identified from contributing practices, which constitutes 5.5% of the total patient cohort. Information on all appointments made by diabetic patients, regardless of the reason, during a 3-year period (2012–2014) was extracted, resulting in 4,474,943 consultations.

### Exposure data

Gridded daily mean temperature datasets for the UK at 5 × 5 km resolution, previously created by the Met Office with financial support from DEFRA, were used to represent exposure for each patient at the time of consultation (http://www.metoffice.gov.uk/climatechange/science/monitoring/ukcp09), with the most recent years of data being provided via the MEDMI project (https://www.data-mashup.org.uk/). The datasets are based on observations from Met Office fixed monitoring stations, with regression and interpolation methods used to generate values on a regular grid, taking into account factors such as latitude and longitude, altitude and terrain shape, coastal influence, and urban land-use.

### Linkage

The patient’s home address at the time of each GP consultation was linked to the gridded temperature dataset. Grid coordinates were converted into the Eastings and Northings coordinate system to allow linkage to sector-level patient postcode data omitting the final 2 letters of the full postcode for confidentiality reasons (i.e. AB12 3). Temperature data were rounded to the nearest 0.5 °C in order to reduce the number of unique combinations of temperatures that may allow potential re-identification of individual patients. To further anonymise the dataset, coordinates were randomised whilst still allowing linkage to the ResearchOne data. This step was necessary to ensure that the simple knowledge that the Met Office dataset is arranged in a fixed 180 rows × 290 columns grid ‘map’ could not be used to geographically re-identify patients. Postcodes for several known geographical locations were checked manually to ensure the randomisation process had been successful. To maintain maximum confidentiality, the data linkage was conducted in-house by the ResearchOne team.

### Analysis

To assess whether temperature exposure influences the timing of GP consultations by diabetic patients we used a fixed-stratum case-crossover approach [10]. With this design, each case is represented by exposure conditions on the day of consultation, and controls by exposures on proximate days. Each consultation event was stratified into a risk-set of 28 days beginning on 1st January 2012. If patients had more than one consultation per day then only the first occurrence was analysed since subsequent consultations are likely to be related.

Conditional logistic regression was then used to assess the association between each event and its control days within that risk-set – traditional confounding factors that are time-invariant over 28-day periods are therefore implicitly adjusted for. Within each risk-set, the case day was matched on the day of the week to its control days. This adjusts for any confounding effect of variation in consultation numbers by day-of-week, but also removes possible autocorrelation in the data, i.e. the increased likelihood of consulting again on subsequent neighbouring days [11]. There were 3 control days for each of the 4,474,943 consultations assessed, with the control days randomly occurring either before or after the case day, or a mixture of the two.

The functional form of the relationship between temperature and odds of GP consultation was first visualised using natural cubic spline functions with interior knots at 0, 10 and 20 °C. This indicated linear increases in the consultation odds both above a high temperature threshold of 22 °C and below a low temperature threshold of 0 °C (Additional file 1: Figure S2). Heat and cold effects were assessed simultaneously. In order to capture possible lagged effects of temperature, and the delays inherent in arranging a GP appointment, distributed lags up to 7 days following exposure were considered. Lags longer than 7 days were also assessed in relation to cold risk but none were observed. For events occurring at the start of each 28-day risk period, data from previous days were complete since exposure was characterized from complete time-series of the temperature values before stratification into non-contiguous periods.

Based on prior hypotheses, assessment was made of the possible modification of heat effects by patient characteristics (age and sex) and by comorbidities (cardiovascular diseases, respiratory diseases, renal failure, and neuropathy), with this being determined by each patient’s consultation history for these conditions. Multiple comorbidities were not considered simultaneously due to power limitations. Increased odds due to medication use was also explored using prescription information for each patient. Based on current knowledge and where at least 5% of cases had been prescribed the treatment at any time during the study period, the medication groups considered were diuretics, salicylates, anticholinergics, antipsychotics and antidepressants. The groups were defined using chapters and subchapters of the British National Formulary Classification system (https://www.bnf.org/). These agents are not specific to diabetes treatment but may pose a risk by affecting skin blood flow or sweating [7, 12].

Associations are presented as odds ratios (OR) and (95% confidence intervals) per 1 °C increase (or decrease) in temperature above (or below) thresholds. Analyses were conducted in STATA14 using the high-power computing facilities at the London School of Hygiene & Tropical Medicine.

## Results

**Fig. 1:**
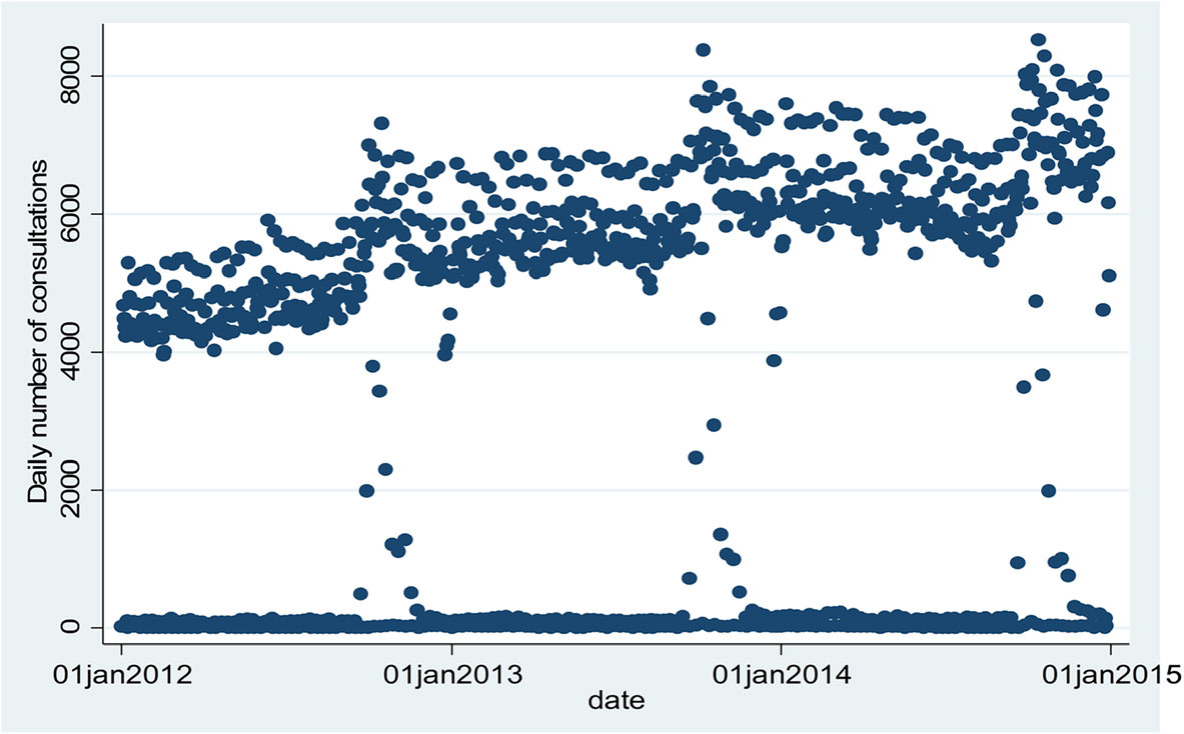
Time-series of daily number of consultations by diabetic patients in participating practices, 2012–2014

Figure 1 displays the daily number of consultations over the 3-year study period. The graph displays a banding of counts by the day of the week, an Autumn peak each year, and a trend of increasing counts - likely due to more practices being recruited onto the database over time. Overall, 52.6% of the patients were male and 57.5% were aged 65 years or older. The mean, minimum and maximum values of daily mean temperature recorded in contributing grid cells during the 3 year study-period were 9.2, −10.5, and 27 °C respectively. The mean, minimum and maximum values recorded specifically on GP consultation days were 10.3, −8.0, and 25.5 °C respectively.

**Fig. 2:**
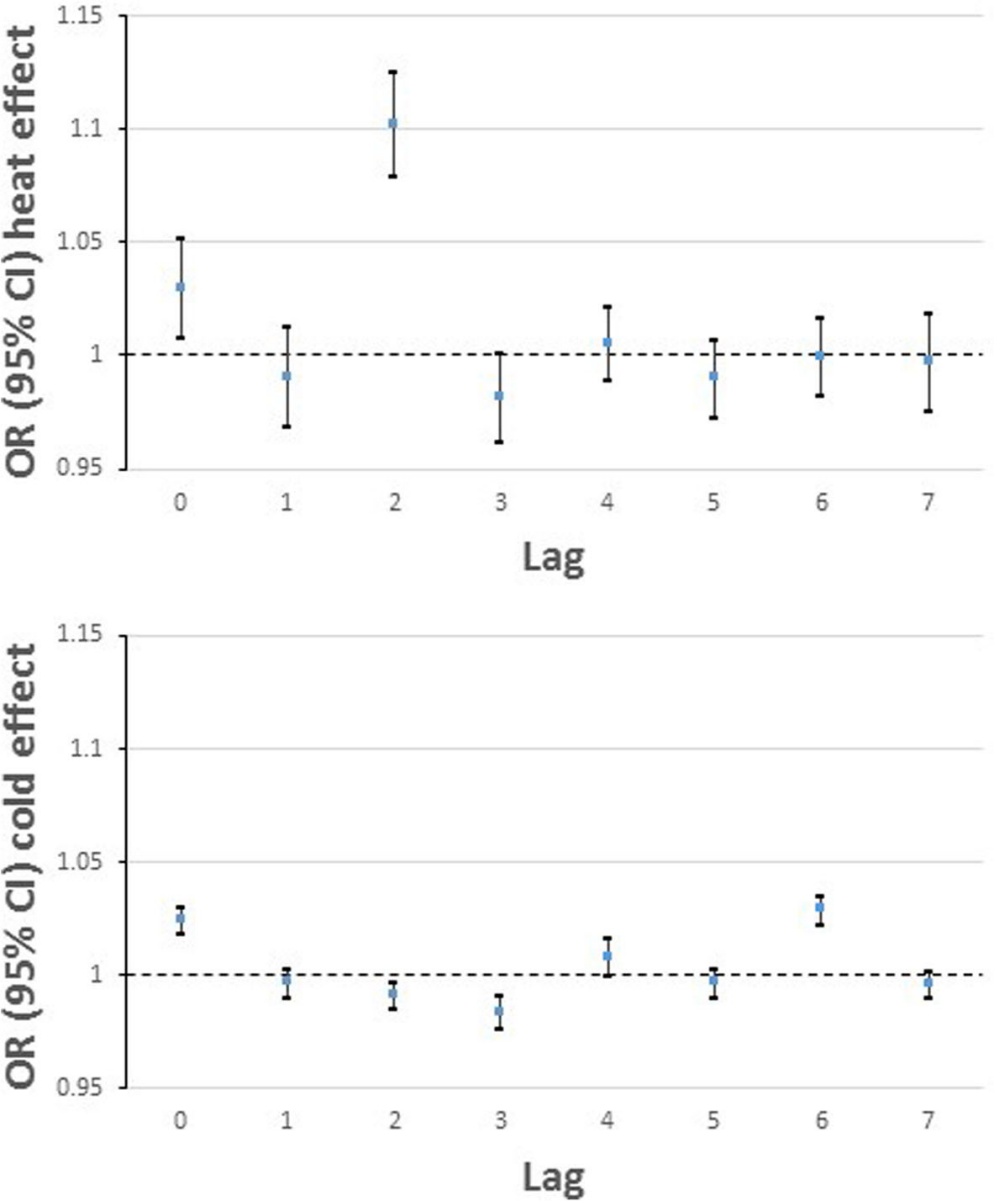
Odds ratios of heat- and cold-related consultations per 1 °C temperature change at individual lags

The odds of a heat-related GP consultation was estimated to be OR = 1.097 (95% CI 1.041, 1.156), indicating a 9.7% increase in odds of consultation for every 1 °C increase in temperature above 22 °C summed for lags 0–7 days from the onset of the temperature rise. Figure 2 shows the distribution of these effects for the separate lags. With heat exposure, the majority of this effect occurred with a 2-day delay: OR = 1.103 (1.080, 1.126). The only other lag that was significantly associated was on the day of exposure (lag 0): OR = 1.030 (1.009, 1.052). The effect of low temperature was smaller but still significantly raised: OR = 1.024 (1.019, 1.030) per 1 °C drop in temperature below 0 °C, summed for lags 0–7 days. Cold risk was significantly raised on lags 0 and 6 days and significantly negative on lags 2 and 3 days. Given the smaller cold effect, and prior hypotheses, subgroup analysis is presented for heat risk only.

Table 1 shows heat effects by patient characteristics and comorbidities. Although interactions did not reach conventional levels of statistical significance, patients aged 65 years and above appeared to be at greater risk than those under 65. Diabetics with respiratory problems were also at greater risk, but the largest differentials were among those with cardiovascular diseases (CVD) who had an almost threefold odds compared to those without CVD complications. Surprisingly, there was no additional risk among diabetics with renal failure nor those with neuropathy; indeed, this group had a reduced odds of consulting on a hot day, although the wide confidence interval limits interpretation.

**Table 1:** Effect modification of heat-related consultation by patient characteristics and comorbidities. OR shown for 1 °C increase above 22 °C and summed for lags 0–7 days

Modifying factor	Number of consultations (%)	OR (95% CI)	*P*-value for interaction
Sex			
Male	2,347,986 (52.6)	1.101 (1.024, 1.185)	0.85
Female	2,113,324 (47.4)	1.090 (1.011, 1.176)	
Age-group			
< 65 years	1,895,277 (42.5)	1.072 (0.991, 1.158)	0.44
65+ years	2,566,034 (57.5)	1.118 (1.040, 1.201)	
CVD			
No	3,611,128 (80.9)	1.066 (1.006, 1.129)	0.19
Yes	851,693 (19.1)	1.171 (1.031, 1.331)	
Respiratory diseases			
No	3,081,816 (69.1)	1.067 (1.002, 1.136)	0.40
Yes	1,381,005 (30.9)	1.122 (1.017, 1.236)	
Renal failure			
No	4,336,129 (97.2)	1.082 (1.026, 1.142)	0.98
Yes	126,692 (2.8)	1.087 (0.776, 1.522)	
Neuropathy			
No	4,112,265 (92.2)	1.097 (1.038, 1.159)	0.09
Yes	350,556 (7.9)	0.923 (0.761, 1.120)	

Although those with CVD comorbidities were slightly older than other patients, age was not the explanation for the highly raised odds among diabetics with CVD complications. The large differentials in heat risk were present only in the relatively younger age-groups (Table 2). Among those aged 75+ years, the odds of heat-related consultation was lower in those patients with CVD comorbidities.

**Table 2:** OR of heat-related consultation, by age-group and CVD comorbidity

	OR (95% CI) per 1 °C increase above 22 °C	
	Diabetics with no CVD comorbidity	Diabetics with CVD comorbidity
Age-group		
0–64 years	1.042 (0.958, 1.134)	1.209 (0.987, 1.480)
65–74 years	1.063 (0.950, 1.190)	1.238 (0.977, 1.568)
75+ years	1.109 (0.989, 1.242)	1.054 (0.839, 1.324)

The odds of consultation on hot days among diabetics taking salicylates was higher compared to those not taking them, although only slightly and not significantly so: 1.113 (0.802, 1.546) vs 1.082 (1.026, 1.142) (Table 3).

**Table 3:** OR of heat-related consultation, by medication use

		OR (95% CI) per 1 °C increase above 22 °C		
		All diabetics	Diabetics with no CVD comorbidity	Diabetics with CVD comorbidity
Medication use				
Diuretics	NO	1.095 (1.013, 1.183)	1.074 (0.988, 1.167)	1.245 (1.003, 1.546)
	YES	1.073 (0.999, 1.153)	1.057 (0.975, 1.146)	1.132 (0.967, 1.326)
Salicylates	NO	1.082 (1.026, 1.142)	1.066 (1.005, 1.130)	1.169 (1.027, 1.331)
	YES	1.113 (0.802, 1.546)	1.071 (0.739, 1.553)	1.147 (0.561, 2.344)
Anticholinergics	NO	1.095 (1.037, 1.157)	1.079 (1.015, 1.146)	1.179 (1.033, 1.347)
	YES	0.944 (0.781, 1.143)*	0.910 (0.737, 1.123)*	1.057 (0.670, 1.666)
Antipsychotics	NO	1.083 (1.025, 1.144)	1.063 (1.001, 1.129)	1.183 (1.038, 1.348)
	YES	1.087 (0.889, 1.330)	1.104 (0.891, 1.369)	0.958 (0.532, 1.724)
Antidepressants	NO	1.094 (1.025, 1.168)	1.070 (0.996, 1.149)	1.230 (1.048, 1.443)
	YES	1.062 (0.971, 1.162)	1.057 (0.957, 1.167)	1.077 (0.872, 1.329)

* *p* < 0.15 for interaction term

Contrary to expectations, diuretic use was associated with reduced odds of a heat-related consultation compared to non-use. The reduction in risk was especially marked in diabetic patients with CVD comorbidity: OR = 1.245 (1.003, 1.546) in non-diuretic users vs 1.132 (0.967, 1.326) in users, although this difference was not statistically significant. A similar pattern was observed with anticholinergic, antipsychotic and antidepressant use, with higher odds for non-users with CVD comorbidity compared to users, but again no interactions reached statistical significance.

## Discussion

Our results show that diabetic patients are at increased odds of consulting a GP during days of temperature extremes, especially during hot weather. Although there was limited statistical power to detect interactions, diabetics with cardiovascular or respiratory comorbidities had an elevated risk of heat-related GP consultation, but there was no evidence for a heightened risk among those with renal failure or neuropathy.

Although some studies have previously reported that cold is associated with increases in GP visits from respiratory infections in the UK and elsewhere [13, 14], to date there is very little evidence on the impacts of high temperatures in the primary care setting, especially among high risk individuals [15, 16]. Most recently, primary care visits for asthma by children were shown to be elevated during summer months in Japan [17]; and in the UK population a spike in GP activity was observed during an individual heat-wave period using syndromic surveillance data [18]. Although the present study did not consider heat impacts on all primary care consultations, our results are likely to be specific to diabetic patients since previous work observed no increased risk of consultation among other patient groups during the 2013 heat-wave [19]. Our study reveals that heat-related GP consultations are apparent in high-risk individuals such as diabetics and are not restricted to extreme heat periods only. Adverse impacts became apparent at fairly moderate daily mean temperature values of 22 °C.

We estimated a 9.7% increase in the odds of a GP consultation per 1 °C rise in high temperatures among type-2 diabetics in England, with the majority of this effect occurring with a 2 day lag – possibly reflecting the time taken to access a GP rather than a biological delay of response to exposure. This increase is greater than that observed for heat-related emergency hospitalisations due to diabetes in high-income settings [20–22], but lower compared to diabetic outpatient visits in a middle-income country with a tropical climate [23]. Heat-waves in Australia had a much greater impact on diabetes mortality compared to diabetes hospital admissions [24]. In our study, the consultation odds increased to over 17% for diabetics with CVD comorbidities. This is consistent with a study from Toronto where heat-related emergency room visits for CVD were particularly elevated in patients with diabetes comorbidity [25]. This indicates that individuals with either diabetes or CVD problems are likely to be at increased risk of heat-illness, but especially so for those with a combination of the two.

Since some medications can interfere with hydration status, sweat production and electrolyte balance, drug-use is commonly cited as increasing the risk of heat-related illness [7]. The classes of drugs considered to be most problematic are often listed in public health guidance documentation available as part of heat protection plans of many countries. However, such advice tends to be based on hypothesized action or on experimental evidence conducted in non high-risk individuals subjected to brief periods of heat exposure that do not replicate conditions of an urban heat-wave [8]. The epidemiologic evidence is very limited – one study observed that use of antipsychotic or hypnotic/anxiolytic medications elevated the risk of heat-related death in people with mental illnesses [26]. A meta-analysis of 4 studies estimated that taking psychotropic medications elevated the risk of death almost twofold during heat-waves [27]. Case reports have linked both older (chlorpromazine) and newer (zuclopenthixol, quetiapine) antipsychotic agents, as well as anticholinergic agents such as benztropine used to treat the Parkinsonian side-effects of antipsychotics, in fatal cases of heat-stroke [28, 29].

However, it is unclear in many previous studies how much of the raised heat-risk was due to the medication use or to the underlying diseases being treated. We were able to disentangle the two factors to some extent by assessing the risk of medication-use in diabetic patients both with and without comorbidities. We observed no evidence of increased heat-risk due to medication use – indeed odds of heat-related consultation appeared to be (non-significantly) lower among diabetics using diuretics, anticholinergics, antipsychotics or antidepressants, especially for those with CVD comorbidities. This agrees with a comprehensive review of the effects of hyperthermia on pharmacokinetics which concluded that interactions between heat exposure and drug therapy are rare and probably limited to special situations in which local blood flow is greatly enhanced [30]. In France, serious metabolic adverse drug reactions (ADRs) among the elderly were *less* frequent in the hot summers of 2003 and 2006 compared to other summers, and other types of ADR showed no variations [31]. One possibility is that diuretics, anticholinergics, antipsychotic and antidepressant agents interfere with the perception of heat and thus alter the threshold for presentation. This could lead to vulnerable individuals on medication bypassing primary care and presenting directly to hospital, however a recent study observed that anticholinergic drug-use in older adults was associated with longer length of stay in hospital during non heat-wave periods but not during heat-wave periods [32].

A major strength of the study is the large number of events analysed. A cohort of 191,842 diabetic patients was identified from a database of GP practices, resulting in over 4 million consultations that were matched to localised temperature conditions around the time of each visit. The use of gridded temperature data allowed for reliable characterisation of exposure for each patient across England, even in remote parts of the country that may be situated some distance from fixed monitoring stations. Use of gridded data also eliminates missing values in exposure.

One potential limitation of analysis is that no other meteorological factors, such as relative humidity, or air pollutants were assessed as potential confounders or effect-modifiers. The role of relative humidity has been shown to be minor in comparison to temperature in the UK [33], and some have argued that pollution should not be a confounder in any relationship between temperature and health [34]. In previous studies on diabetes outcomes where results are reported both with and without air pollution control, the heat effect was not reduced after pollutant adjustment [21, 35]. One drawback with the health data is that, as with other clinical computing systems, TPP has under-representation in some parts of the country, in this case the South East and East of England regions, which may limit generalisability [36]. Furthermore, we were unable to distinguish between emergency GP consultations from elective ones; the latter we would not expect to be associated with environmental triggers, however this should only serve to introduce noise into our data and bias estimated odds ratios towards unity. Effects may also have been attenuated by use of broad diagnoses for diabetes (Additional file 1: Table S1) and comorbidities, and so further work should consider more specific inclusion codes. We considered consultations from any cause since heat and cold exposure can aggravate ill-health from multiple causes, although such environmental factors are very unlikely to be indicated in the medical records. Consultations from specific causes may yield stronger associations, although lack of power may be a limitation. Also, although medication use was determined from the prescription information detailed for each patient, we did not consider the frequency of use due to lack of power. Furthermore, it was impossible to know whether treatment protocols were adhered to, and so non-adherence may be an alternative explanation for the lack of modifying effect observed with drug use.

Our work highlights the potentially important role that GPs can play in mitigating heat burdens. As well as heat impacts being apparent at the primary care level for diabetics, GP visits are one of the few social interactions that vulnerable individuals may experience [37]. When hot weather is forecast, timely medical advice either during routine appointments or during home visits could minimize the risk of heat stress. Although in the current study we were unable to follow-up patients to determine the outcome of each consultation, simple heat protection advice and management strategies by the GP have the potential to prevent subsequent heat-related GP visits and further deterioration possibly resulting in hospitalizations and deaths. However, for such actions to be effective those most vulnerable during hot weather need to be identifiable and the advice and interventions offered need to be based on sound evidence. Diabetic patients, especially those with CVD comorbidities, should be considered at high-risk and therefore require appropriate management during temperature extremes, although diabetic patients that are physically active and have good blood glucose control may tolerate heat as well as healthy individuals [7]. Drug use is often cited as a risk factor for heat-illness in public health protection plans and treatment regime adjustments during hot weather are commonly advocated, however our study suggests that such advice is likely to be incorrect for diabetic patients in particular, and this may be the case for other patients too. Similar assessment of medication effects on risks of heat exposure in patients with other diseases should be the subject of future research. The current work could also be extended by considering the primary reason for GP consultation and whether this varied by temperature, and also assessment of which mechanisms in diabetic patients with cardiovascular diseases raise the risk of heat exposure. More work is also needed on measuring the effectiveness of specific advice concerning how to minimise heat stress in the primary care setting.

## Conclusions

Consultations with primary care in people with type-2 diabetes are increased during temperature extremes, particularly on hot days, with suggestive but not definitive evidence that those with CVD complications are at higher risk. Public health protection measures should provide advice to patients with type-2 diabetes and their healthcare providers about dealing with heat exposure, and the contribution that GPs could make in helping identify and manage such patients should be enhanced. The commonly proffered instruction that certain medication use heightens the risk of heat-illness was not borne-out by our study, and such advice may need to be reconsidered in public health heat protection plans.

## Additional file

Additional file 1: Table S1. Read codes and descriptions. **Figure S2.** Relationship between temperature and odds of consultation. (DOCX 65 kb)
