# Supplementary material for: The effect of ambient temperature on type-2-diabetes: case-crossover analysis of 4+ million GP consultations across England
Source: Environ Health. 2017 Jul 12;16:73. doi: 10.1186/s12940-017-0284-7 (PMC5506566; doi:10.1186/s12940-017-0284-7)
Supplement: Additional file 1: Table S1. — Read codes and descriptions. Figure S2. Relationship between temperature and odds of consultation. (DOCX 65 kb) [file 12940_2017_284_MOESM1_ESM.docx]

***Supplemental***

*S1: Read codes and descriptions*

| \| XaMhK \| Yapiu \| Diabetes type 2 review \| \| \| \| --- \| --- \| --- \| --- \| --- \| \| X40J5 \| Y41PZ \| Type 2 diabetes mellitus \| \| \| \|  \| C1090 \| YagvQ \| Type II diabetes mellitus with renal complications \| \| \|  \|  \| XaF05 \| Yagvz \| Type II diabetes mellitus with nephropathy \| \|  \|  \| XaIzQ \| YaluR \| Type II diabetes mellitus with persistent proteinuria \| \|  \|  \| XaIzR \| YaluT \| Type II diabetes mellitus with persistent microalbuminuria \| \|  \| C1091 \| YagvR \| Type II diabetes mellitus with ophthalmic complications \| \| \|  \|  \| C1096 \| Yagvt \| Type II diabetes mellitus with retinopathy \| \|  \|  \| XaFmA \| YagvW \| Type II diabetes mellitus with diabetic cataract \| \|  \|  \| XaJQp \| YamWw \| Type II diabetes mellitus with exudative maculopathy \| \|  \| C1092 \| YagvS \| Type II diabetes mellitus with neurological complications \| \| \|  \|  \| XaEnp \| Yagvw \| Type II diabetes mellitus with mononeuropathy \| \|  \|  \| XaEnq \| Yagvy \| Type II diabetes mellitus with polyneuropathy \| \|  \|  \| XaKyX \| Yatx4 \| Type II diabetes mellitus with gastroparesis \| \|  \| C1093 \| YagvT \| Type II diabetes mellitus with multiple complications \| \| \|  \| C1094 \| YagvU \| Type II diabetes mellitus with ulcer \| \| \|  \| C1095 \| YagvV \| Type II diabetes mellitus with gangrene \| \| \|  \| C1097 \| Yagvu \| Type II diabetes mellitus - poor control \| \| \|  \| L1806 \| YMAow \| Pre-existing diabetes mellitus, non-insulin-dependent \| \| \|  \| XaELQ \| Yagvv \| Type II diabetes mellitus without complication \| \| \|  \| XaFWI \| Yagw0 \| Type II diabetes mellitus with hypoglycaemic coma \| \| \|  \| XaFn7 \| Yagwv \| Type II diabetes mellitus with peripheral angiopathy \| \| \|  \| XaFn8 \| Yagwx \| Type II diabetes mellitus with arthropathy \| \| \|  \| XaFn9 \| Yagwz \|  \| Type II diabetes mellitus with neuropathic arthropathy \| \| XaIfG \| YalAM \| Type 2 diabetes on insulin \| \| \| \| XaIfI \| YalAO \| Type 2 diabetes on diet only \| \| \| \| XaXZR \| YauMu \| H/O: diabetes mellitus type 2 \| \| \| \| XaXZR \| YauMw \| History of diabetes mellitus type 2 \| \| \| \| C1094 \| YajRb \| Type 2 diabetes mellitus with ulcer \| \| \| \| Xaagf \| YawW8 \| Type 2 diabetes mellitus in remission \| \| \| \| C1095 \| YajRc \| Type 2 diabetes mellitus with gangrene \| \| \| \| C1097 \| YajRe \| Type 2 diabetes mellitus - poor control \| \| \| \| X40JJ \| Y41Q3 \| Maturity onset diabetes in youth type 2 \| \| \| \| X40J6 \| Yakg3 \| Insulin treated Type 2 diabetes mellitus \| \| \| \| XaFn8 \| YajRn \| Type 2 diabetes mellitus with arthropathy \| \| \| \| XaFn9 \| Yagwz \|  \| Type II diabetes mellitus with neuropathic arthropathy \| \| \| XaF05 \| YajRj \| Type 2 diabetes mellitus with nephropathy \| \| \| \| C1096 \| YajRd \| Type 2 diabetes mellitus with retinopathy \| \| \| \| XaVw4 \| YatlS \| Family history of diabetes mellitus type 2 \| \| \| \| C1011 \| YaluU \| Type 2 diabetes mellitus with ketoacidosis \| \| \| \| XaKyX \| Yao4G \| Type 2 diabetes mellitus with gastroparesis \| \| \| \| X40JJ \| Yal7O \| Diabetes mellitus autosomal dominant type 2 \| \| \| \| XaEnq \| YajRi \| Type 2 diabetes mellitus with polyneuropathy \| \| \| \| XaEnp \| YajRh \| Type 2 diabetes mellitus with mononeuropathy \| \| \| \| XaELQ \| YajRg \| Type 2 diabetes mellitus without complication \| \| \| \| X40JJ \| Y41Q2 \| MODY - Maturity onset diabetes in youth type 2 \| \| \| \| XaFmA \| YajRl \| Type 2 diabetes mellitus with diabetic cataract \| \| \| \| C1031 \| YaluW \| Type 2 diabetes mellitus with ketoacidotic coma \| \| \| \| XaFWI \| YajRk \| Type 2 diabetes mellitus with hypoglycaemic coma \| \| \| \| C1090 \| YajRS \| Type 2 diabetes mellitus with renal complications \| \| \| \|  \| XaF05 \| Yagvz \| Type II diabetes mellitus with nephropathy \| \| \|  \| XaIzQ \| YaluR \| Type II diabetes mellitus with persistent proteinuria \| \| \|  \| XaIzR \| YaluT \| Type II diabetes mellitus with persistent microalbuminuria \| \| \| XaJQp \| YamRz \| Type 2 diabetes mellitus with exudative maculopathy \| \| \| \| XaFn7 \| YajRm \| Type 2 diabetes mellitus with peripheral angiopathy \| \| \| \| C1093 \| YajRY \| Type 2 diabetes mellitus with multiple complications \| \| \| \| XaIzQ \| YaluQ \| Type 2 diabetes mellitus with persistent proteinuria \| \| \| \| XaFn9 \| YajRo \| Type 2 diabetes mellitus with neuropathic arthropathy \| \| \| \| C1091 \| YajRT \| Type 2 diabetes mellitus with ophthalmic complications \| \| \| \|  \| C1096 \| Yagvt \| Type II diabetes mellitus with retinopathy \| \| \|  \| XaFmA \| YagvW \| Type II diabetes mellitus with diabetic cataract \| \| \|  \| XaJQp \| YamWw \| Type II diabetes mellitus with exudative maculopathy \| \| \| C1092 \| YajRU \| Type 2 diabetes mellitus with neurological complications \| \| \| \|  \| XaEnp \| Yagvw \| Type II diabetes mellitus with mononeuropathy \| \| \|  \| XaEnq \| Yagvy \| Type II diabetes mellitus with polyneuropathy \| \| \|  \| XaKyX \| Yatx4 \| Type II diabetes mellitus with gastroparesis \| \| \| XaIzR \| YaluS \| Type 2 diabetes mellitus with persistent microalbuminuria \| \| \| \| XaIrf \| YalWY \| Hyperosmolar non-ketotic state in type 2 diabetes mellitus \| \| \| |
| --- | --- | --- | --- | --- | --- | --- | --- | --- | --- | --- | --- | --- | --- | --- | --- | --- | --- | --- | --- | --- | --- | --- | --- | --- | --- | --- | --- | --- | --- | --- | --- | --- | --- | --- | --- | --- | --- | --- | --- | --- | --- | --- | --- | --- | --- | --- | --- | --- | --- | --- | --- | --- | --- | --- | --- | --- | --- | --- | --- | --- | --- | --- | --- | --- | --- | --- | --- | --- | --- | --- | --- | --- | --- | --- | --- | --- | --- | --- | --- | --- | --- | --- | --- | --- | --- | --- | --- | --- | --- | --- | --- | --- | --- | --- | --- | --- | --- | --- | --- | --- | --- | --- | --- | --- | --- | --- | --- | --- | --- | --- | --- | --- | --- | --- | --- | --- | --- | --- | --- | --- | --- | --- | --- | --- | --- | --- | --- | --- | --- | --- | --- | --- | --- | --- | --- | --- | --- | --- | --- | --- | --- | --- | --- | --- | --- | --- | --- | --- | --- | --- | --- | --- | --- | --- | --- | --- | --- | --- | --- | --- | --- | --- | --- | --- | --- | --- | --- | --- | --- | --- | --- | --- | --- | --- | --- | --- | --- | --- | --- | --- | --- | --- | --- | --- | --- | --- | --- | --- | --- | --- | --- | --- | --- | --- | --- | --- | --- | --- | --- | --- | --- | --- | --- | --- | --- | --- | --- | --- | --- | --- | --- | --- | --- | --- | --- | --- | --- | --- | --- | --- | --- | --- | --- | --- | --- | --- | --- | --- | --- | --- | --- | --- | --- | --- | --- | --- | --- | --- | --- | --- | --- | --- | --- | --- | --- | --- | --- | --- | --- | --- | --- | --- | --- | --- | --- | --- | --- | --- | --- | --- | --- | --- | --- | --- | --- | --- | --- | --- | --- | --- | --- | --- | --- | --- | --- | --- | --- | --- | --- | --- | --- | --- | --- | --- | --- | --- | --- | --- | --- | --- | --- | --- | --- | --- | --- | --- | --- | --- | --- | --- | --- | --- | --- | --- | --- | --- | --- | --- | --- | --- | --- | --- | --- | --- | --- | --- | --- | --- | --- | --- | --- | --- | --- | --- | --- | --- | --- | --- | --- | --- | --- | --- | --- | --- | --- | --- | --- | --- | --- | --- |

*S2: Relationship between temperature and odds of consultation*
